# Clinical Correlation and Role of Cyclin D1 Expression in Glioblastoma Patients: A Study From North India

**DOI:** 10.7759/cureus.22346

**Published:** 2022-02-17

**Authors:** Mamta Jaiswal, Archana Tripathi, Dezy Singh, Arvind Kumar, Monika Singh, Neha Batra, Anil Verma

**Affiliations:** 1 Pathology, Guru Shri Gorakhnath Chikitsalaya, Gorakhpur, IND; 2 Pathology, Baba Raghav Das Medical College, Gorakhpur, IND; 3 Forensic Medicine • Toxicology, Uttarakhand Ayurved University, Rishikesh, IND; 4 Pathology, All India Institute of Medical Sciences, Rishikesh, IND; 5 Pathology, Government Doon Medical College, Dehradun, IND; 6 Pathology, Raipur Institute of Medical Sciences, Raipur, IND

**Keywords:** north india, high mortality, high grade gliomas, midline high grade glioma, immunohistochemistry staining, glioblastoma multiforme, cyclin d1

## Abstract

Background/Aims

Glioblastoma multiforme (GBM) is the most aggressive primary brain tumor. Cyclin D1 is a protein that in humans is encoded by the CCND1 gene. Cyclin D1 protein is frequently overexpressed in malignant gliomas.

Methods

It is an observational study comprising 40 biopsy-proven cases of GBM in a span of one and half years. Immunohistochemistry (IHC) was used with Cyclin D1 monoclonal antibody. Cyclin D1 on the outcome was assessed using the Kaplan-Meier survival estimate and compared by log-rank test.

Results

Cyclin D1 was expressed in 60% of patients. The majority (72.5%) of patients expired during the study period, out of which 69% showed immune-expression in contrast to living subjects, out of which only 45.5% of patients exhibited expression. The maximum number of glioblastoma patients were aged between 41 and 50 years (40%), followed by those aged between 31 and 40 years (20%). The male to female ratio of study subjects was 3.44:1.

Conclusion

The study concluded that there is no significant association between Cyclin D1 expression status and different demographic, clinical, and outcome variables.

## Introduction

Glioma is a type of tumor that starts in the brain or spine. It is called a glioma because it arises from glial cells. Glioblastoma multiforme is the most common form of primary malignant tumor of the brain, and it comprises 16% of a primary brain tumor [1]. It also tends to arise from low-grade glial tumors [2]. This tumor can involve any age group, but after the fourth decade, its incidence raises relatively [3]. Clinically, gliomas are divided into four grades; unfortunately, the most aggressive of these, grade 4 or glioblastoma multiforme (GBM), is also the most common in humans. Because most patients with GBMs die of their disease in less than a year and essentially none has long-term survival. Cyclins are synthesized during specific cell cycle phases, and their function is to activate the cyclin-dependent kinases (CDKs). On completion of this task, their level decreases rapidly.

The orderly progression of the cells through the various phases of the cell cycle is orchestrated by CDKs and their inhibitors. In humans, there are three closely related Cyclin D proteins Cyclin D1, Cyclin D2, and cyclin D3. G1/S-specific cyclin-D1 is a protein that in humans is encoded by the CCND1 [4]. This cyclin forms a complex and functions as a regulatory subunit of CDK4 or CDK6, whose activity is required for cell cycle G1/S transition. This protein has been shown to interact with tumor suppressor protein Rb and the expression of this gene is regulated positively by Rb. Mutations, amplification, and overexpression of this gene, which alters cell cycle progression, are observed frequently in various tumors and may contribute to tumorigenesis [5-7]. However, aberrant gene amplification and overexpression of cyclin D1 have been observed in glioma biopsy specimens and a small number of malignant glioma cell lines. A systematic immunohistochemical (IHC) study to evaluate the association between cyclin D1 and survival of Glioblastoma patients, as well as the patterns of glioblastoma as defined in the new WHO Classification (2007), has yet not been performed. In this study, we will try to find out the exact relation between the expression of cyclin D1 and the survival of Glioblastoma patients. If immunohistochemistry plays a significant role in tumor behavior, it will undoubtedly be counted as a patient's primary health care since IHC is a fundamental ancillary technique.

## Materials and methods

It is an observational study hospital-based conducted at the Department of Neurosurgery & Neuropathology division of the Department of Pathology, King George's Medical University, Lucknow, and Dr. Ram Manohar Lohia Institute of Medical Sciences, Lucknow. The study is comprised of biopsy-proven 40 glioblastoma cases histopathologically proven cases of glioblastoma without any history of prior treatment over one and half years. The biopsies & resected specimens were collected in 10% formalin.

A paraffin section was made and stained by routine hematoxylin and eosin (H&E) for histopathological examination of GBM & defined subtypes or variants of GBM. Immunohistochemistry (IHC) for cyclin D1 antigen was done using anti-cyclin D1 primary antibody and secondary antibody, and both were procured from Santa Cruz Biotechnology Inc. The primary antibody (1:100) used was cyclin D1 (DCS-6) mouse monoclonal antibody raised against the recombinant full-length human protein. The secondary antibody used was an m-IgG Fc BP-HRP (Sigma-Aldrich, UK) and counterstained with hematoxylin for 30 seconds.

3μm-thick sections were cut from the paraffin block and placed on 3-aminopropyl triethoxysilane (APES) adhesive slides. These sections were deparaffinized at 60°C for 60 minutes, followed by incubation in xylene and hydration in a series of decreasing ethanol concentrations (100%, 70%, and 30%). The sections were placed in a pressure cooker container filled with antigen retrieval solution (0.01mol/L citrate buffer, pH 6.8) and boiled for 14 minutes for antigen retrieval. The sections were washed in phosphate-buffered saline (PBS, pH 7.6) and immersed in 3% hydrogen peroxide and 100% methanol for 10 min to remove endogenous peroxidase activity. The slides were incubated with primary antibodies at a dilution of 1:50 overnight at 4°C with cyclin D1 (clone FP12) rabbit monoclonal antibody. After washing with PBS, the sections were incubated with biotinylated goat anti-mouse immunoglobulin (Dako Ltd) for 30 min at room temperature, washed with PBS, then incubated with streptavidin-peroxidase conjugate (1:500; Amersham Pharmacia Biotech, Bucks, UK) for 30 min. The sections were developed with diaminobenzidine tetrahydrochloride solution (Sigma-Aldrich, Poole, UK) and 0.1% H_2_O_2_ and counterstained with hematoxylin for 30 seconds. The sections were dehydrated in ascending alcohols, then dried and mounted. At every stage of the staining process, positive and negative controls were set. Immunohistochemical staining was performed using the streptavidin-biotin-HRP complex method of Immunoperoxidase Secondary Detection System (Santa Cruz Biotechnology Inc.) according to the manufacturer’s protocols.

IHC Expression: Nuclei were scored as positive or negative in each slide. Labeling index was defined as the number of positive nuclei per total number of nuclei X 100, after counting at least 1000 cells at high power magnification. The percentage of cyclin D1 Immunopositive tumor cells was counted in 5 consecutive microscopic fields (magnification 100x) per tumor sample in the area with the highest density of these cells. One hundred tumor cell nuclei were evaluated in each field, and the mean for every five fields was calculated. A distinct granular brown nuclear stain was scored as positive. A cutoff value of <5% immunopositive cells was considered negative, and >5% immunopositive cells were considered positive. Cases categorized based on the immune expression of cyclin D1 antibody exhibited by nucleus by the tumor cell. 

A zero grade was given when tumor cell shows less than 5% immunoexpression of total tumor cell, low expression where immunostain shows 5 to 50% expression of tumor cells., high in more than 50% of tumor cell. Statistical tests employed are Mean, Standard Deviation, Chi-square test, Analysis of Variance (ANOVA), ROC Curve, and Level of significance.

## Results

**Figure 1:**
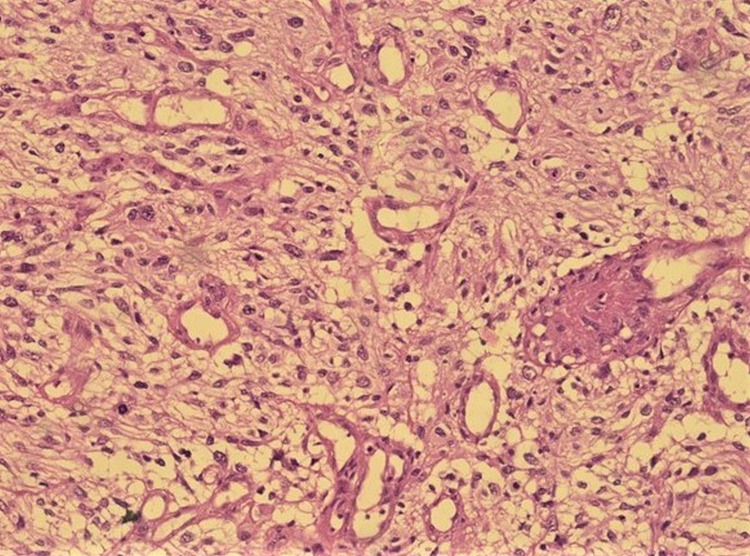
Tumor cells show moderate nuclear pleomorphism with vesicular chromatin, indistinct cytoplasm along microvascular proliferation (H&E 200).

**Figure 2:**
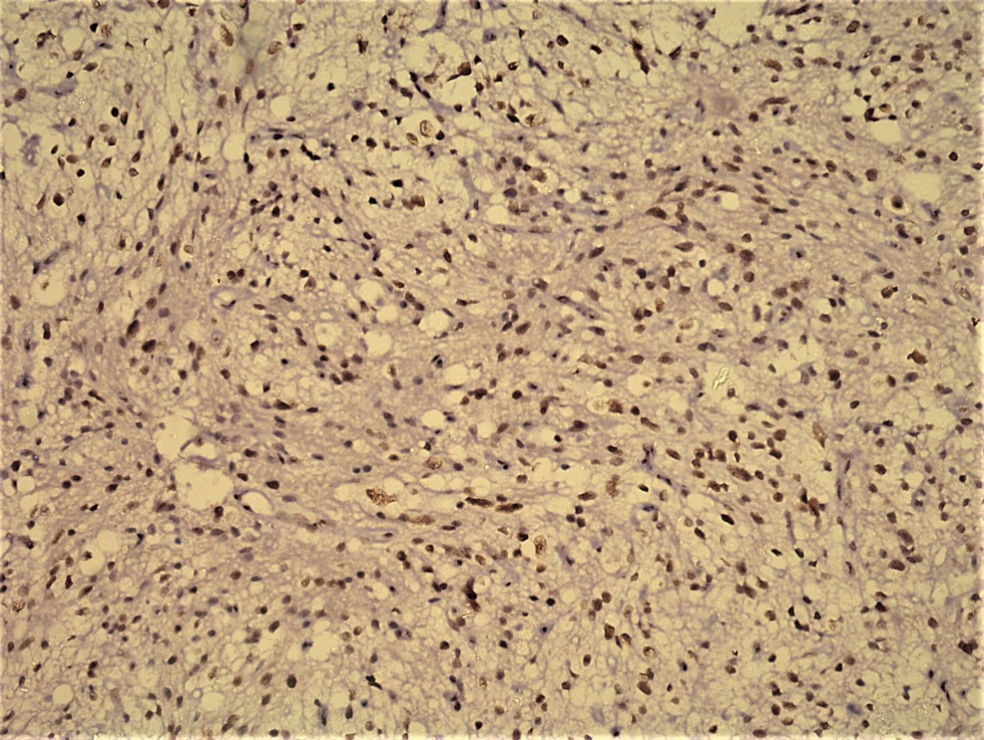
Nuclear CyclinD1 expression in tumor cell (IHC 200x).

A total of 40 cases of glioblastoma were enrolled in the study. The age of cases ranged from 10 years to 69 years. The mean age of patients was 42.20±14.38 years (Median 45 years). A maximum number of patients were aged between 41-50 years (n=16; 40%), followed by those aged between 31-40 years (n=8; 20%). There were only 3 (7.5%) patients aged 21-30 years. A total of 4 (10%) patients each were aged 10-20 and 61-70 years respectively, while a total of 5 (12.5%) patients were aged between 51 to 60 years. Majority of subjects were males (n=31; 77.5%), there were only 9 (22.5%) females. The male to female ratio of study subjects was 3.44:1. A total of 37 (92.5%) patients were GBM alone. There were three patients (7.5%), each having GBM with small cell, Giant cell GBM and Gliosarcoma respectively. The temporal site was the most involved, followed by parietal (25%), frontal (20%), and occipital (20%) parts, respectively. Cyclin D1 was expressed in 24 (60%) patients. The majority of subjects (23) had expression in the range of 5-50%. Sixteen (40%) subjects had no expression, while 1 (2.5%) patient had high expression. (Figure 1, 2).

Although among those who survived, most subjects had no expression as against the majority of subjects who expired showing expression. There was only one case with a high expression level, and that patient expired. Despite these proportional differences, the intergroup difference was not significant statistically (p=0.159) (Table 1).

**Table 1:** Association of Cyclin D1 expression status with mortality of patients. Coefficient of determination (𝑅²)=-3.677, Degree of freedom (DF)=2, p=0. 159, Hi2h= Heterogeneity statistics i2

Level of Expression	Expired (n=29)		Alive (n=ll)		Total
	No.	o/o	No.	o/o	
No expression	9	31.0	7	63.6	16
Low Expression	19	65.5	4	36.4	23
Hi2h Expression	1	3.4	0	0	1

**Figure 3:**
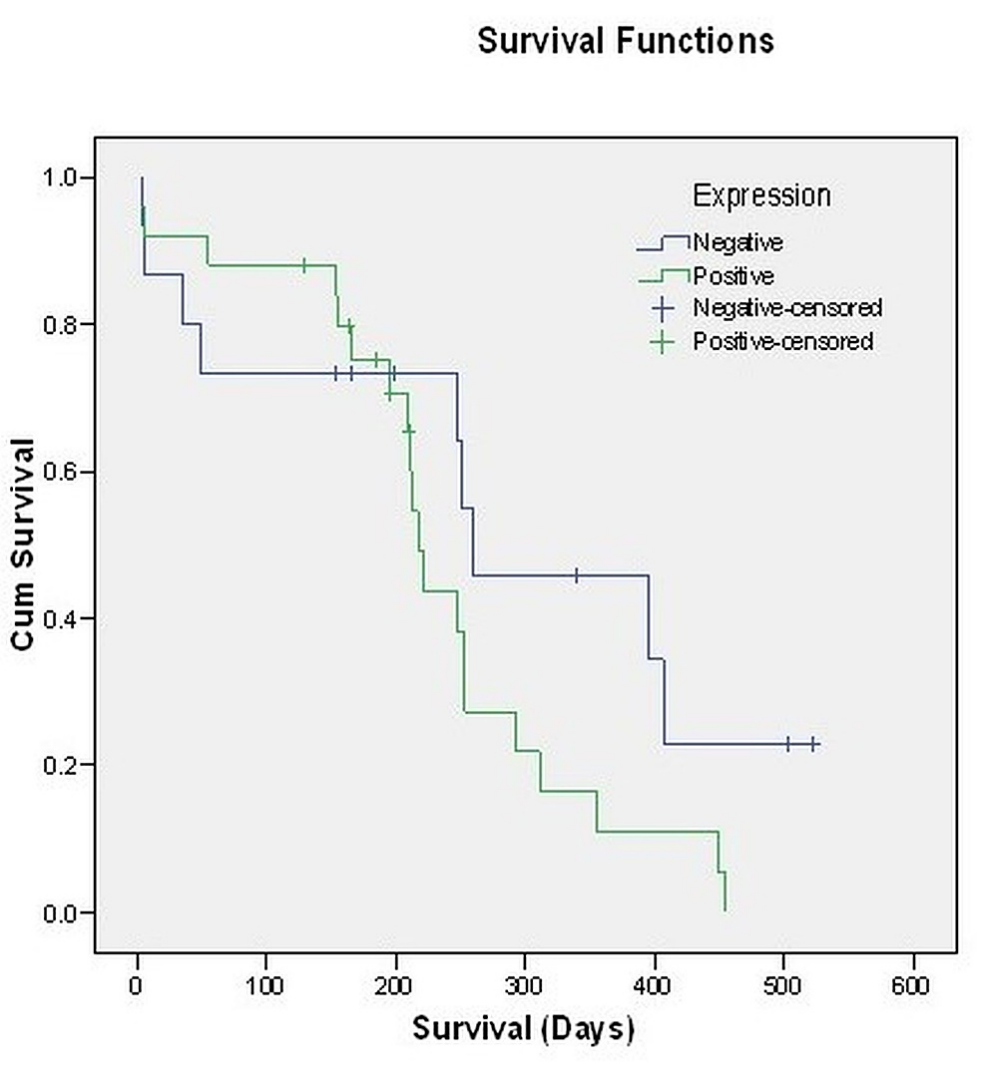
Survival Curve

Inbox plot, it is observed that the 25th percentile value and the minimum value coincided (i.e., more than one-quarter values were 0). One value (66%) has been depicted as an outlier on the plot and excluding this value, the range of expression becomes from 0% to 38% (Figure 3).

No statistically significant association was noted between the expression percentage of Cyclin D1 with various demographic, clinical, and outcome variables (Table 2).

**Table 2:** Association of Cyclin D1 expression status with various demographic, clinical and outcome variables. GBM- Glioblastoma multiforme

SN	Variable	n	No. of patient with expression	% of expression	X^2^	p
Age						
1.	<45 Years	24	14	58.3	0.444	0.505
2.	>45 Years	16	11	68.8		
Gender						
1.	Female	9	6	66.7	0.086	0.769
2.	Male	31	19	61.3		
Diagnosis						
1.	GBM	37	23	62.2	2.868	0.412
2.	GBM with small cell	1	1	100.0		
3.	Giant cell GBM	1	0	0.0		
4.	Gliosarcoma	1	1	100.0		
Survival Status						
1.	Expired	29	20	69.0	1.881	0.170
2.	Alive	11	5	45.5		

The percentage expression of Cyclin D1 ranged from 0 to 66%. The mean expression was 11.85±13.94% (Mean±SD), and the median was 10%. The 5th to 95th percentile range of expression was between 0 to 37.70%. Survival analysis for subjects with different Cyclin D1 expression strata revealed that though the median survival time was higher among those with no Cyclin D1 expression (259.00±102.21 days) as compared to those with Cyclin D1 expression (217.00±6.79 days) yet the difference between the two strata was not significant statistically (p=0.120 - Log Rank - Mantel-Cox test) (Table 3).

**Table 3:** Means and medians for survival time for different expression strata of Cyclin D1.

Expression	Mean Survival Period in Days				Median Survival Period in Days			
	Estimate	Std. Error	95% Confidence interval		Estimate	Std. Error	95% Confidence interval	
			Lower	Upper			Lower	Upper
Negative	287.294	50.759	187.807	386.781	259	102.209	58.67	459.33
Positive	232.335	25.075	183.189	281.482	217	6.787	203.698	230.3
Overall	251.883	24.901	203.076	300.69	248	21.342	206.169	289.831

No association between age, gender, and diagnostic entities with cyclin D1 expression was observed (p>0.05). However, as compared to those who expired the mean expression percentage was significantly higher among those who survived (p=0.035). As a significant association between survival and mean expression percentage was observed, a receiver-operator curve (ROC) analysis was done to find a suitable cutoff to predict the survival outcome based on the expression percentage of Cyclin D1. The area under the curve was observed to be 0.715, which indicated a moderate efficacy of the percentage of Cyclin D1 expression to predict the outcome expiry. A cutoff value >10.50 was observed to be 55.2% sensitive and 81.8% specific (Table 4 and Figure 3).

**Table 4:** Area of Cyclin expression percentage level under the curve for the outcome expiry. The test result variable(s): Cyclin D1 Exp (%) has at least one tie between the positive actual state group and the negative actual state group. Statistics may be biased. a- Under the non-parametric assumption, b-Null hypothesis: true area = 0.5

Area	Standard Error(a)	Asymptotic significance(b)	Asymptotic 95% Confidence Interval	
			Lower	Upper
0.715	0.080	0.038	0.557	0.872

**Figure 4:**
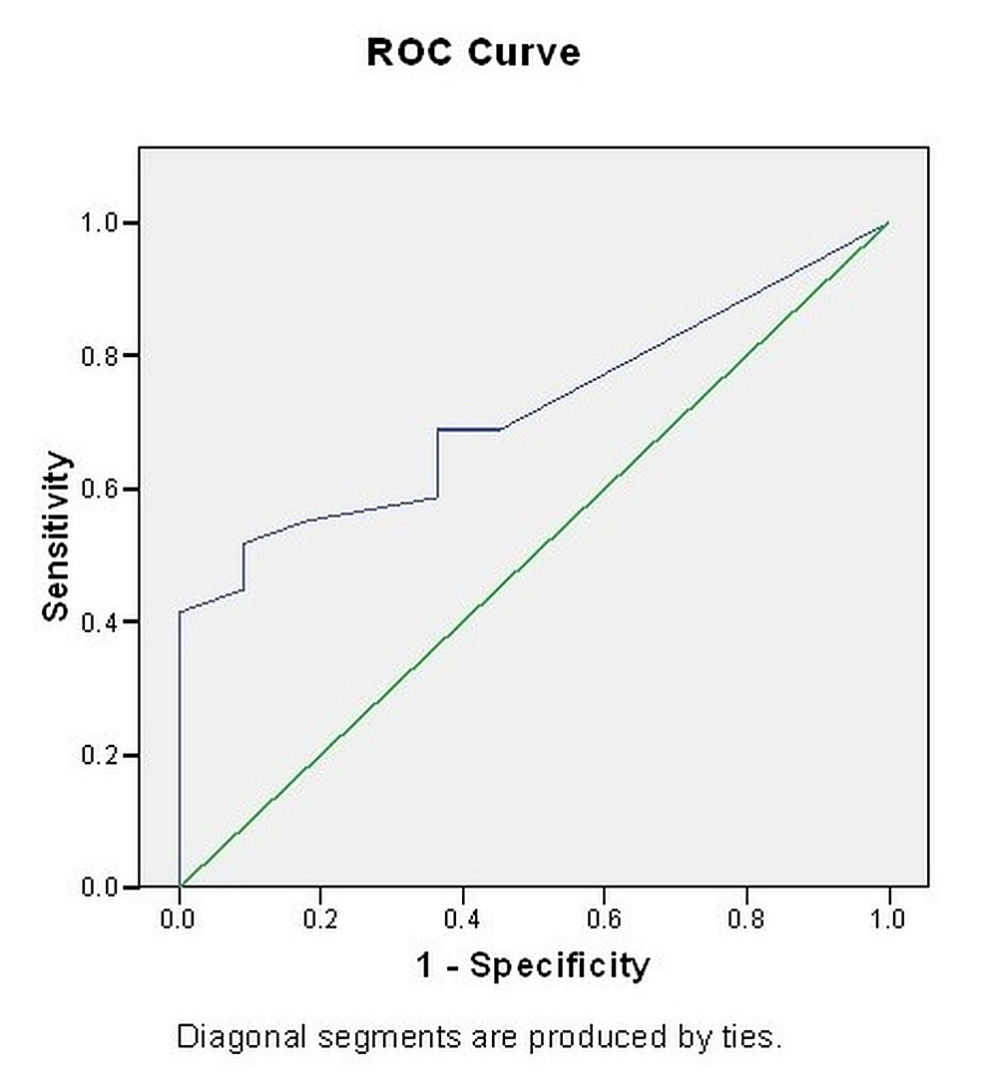
Receiver operating characteristic curve (ROC) curve.

## Discussion

GBM is the most common malignant primary brain tumor making up 54% of all gliomas and 16% primary brain tumors [8]. Overexpression of cyclin D1 was shown in many tumors. Excessive cyclin D1 is an important cause of many tumors [9-14]. However, there are still some controversies about whether the overexpression of cyclin D1 exists in brain gliomas. In our study maximum number of patients (40%) were in the age group of 41 to 50 years, followed by 20% of patients in the range of 31 to 40 years. The prevalence decreased at both extremes of age. In a study by Lantas PL et al. and Ostrom (2002), the peak incidence was in the age group of 45 to 75 years and 75-84 yr respectively [8,15]. This discrepancy may be due to the lower life expectancy in the Indian subcontinent compared to that in the Western world and the reluctance shown to medical consultation by the dependent class of society.

Most of the patients were males with a male: female ratio of 3.44:1, consistent with other studies [8,16]. The higher male prevalence in our study could be due to a still deprived access of women to medical facilities in this part of the country. Thirty-seven patients comprising 92.5% of the study had GBM with classical histology, and its variants comprised only a small percentage (7.5%) of the patients. This finding was in accordance with other studies in the literature. Distant metastasis is rare [17,18], and a craniotomy is said to be responsible for extracranial spreads [19].

Maximum patients had tumors of the temporal region (35%), followed by the parietal region (25%). Twenty percent (20%) of the tumors involved the frontal and the occipital regions. This was in close association with the study by Lee TT et al., 1997 at University Hospital Zurich in which the temporal lobe was involved in 31% of patients, the parietal lobe in 24%, frontal in 23%, and occipital lobe in 16% of the patients [20]. Our study out of the 40-patient cyclin D1 expressed in 24 (60%) patients.

In the present study, most subjects had expressions in the range of >5-50% were considered the low expression, only 2.5% had >50% expression was considered the high expression, and the remaining 40% subject had no expression. Cyclin D1 is a protein encoded by the CCND1 gene and is a cell cycle regulator at the G1-S checkpoint. In our study, cyclin D1 was expressed in 62.5% of patients with GBM. Its mean value among 40 patients was 11.85±13.944, and the median value 10.00. As per the study by Zhang X et al., 2005 cyclin D1 was expressed in 55.8% (29 of 52) gliomas [14]. Biernat W et al. 1997 in their study found expression of cyclin D1 in 50% of GBMs [21].

Biernat W et al. and Mahzouni P et al. showed that Cyclin D1 is a wrong prognostic marker of GBM. Slight staining on IHC for cyclin D1 is found in normal brain and tumors of WHO Grade-1 [22,23]. With increasing histological malignancy, cyclin D1 expression increases markedly. Intense staining is found in high-grade glioma (WHO 3 and 4) and is significantly higher than low-grade gliomas (WHO 1 and 2).

Our findings are similar to those of Ahmad F et al. (2009), who observed staining of that cyclin D1 was highly expressed in benign and low grades gliomas of brain tumors nevertheless decreased in higher grades of tumors [24]. Krtolica et al. has reported similar p27 and cyclin D1 expression pattern in human ovarian carcinoma cells. The study exposed ovarian cancer cells to hypoxia relatively increasing in time [25]. As the time increased, the hypoxic conditions became more dominant with the decreasing availability of oxygen. They monitored the expression pattern of p27 and cyclin D1 and several other proteins according to specific time points. They subsequently found p27 protein elevation in more hypoxic conditions. Nevertheless, they revealed a lowering in cyclin D1 protein expression while the hypoxia became more dominant.

Similarly, Chakrabarty A et al. (1996) found a strong positive correlation between labeling indices for cyclinD1and MIB1 proliferation marker in diffuse astrocytoma [26]. Correlation broke down between anaplastic astrocytoma and glioblastoma. Many other studies showed that the overexpression of cyclin d1 occurs in high-grade astrocytoma [4,27]. However, in our study expression of cyclin D1 is low.

Mahzouni P et al. observed cyclin D1expression only in normal brain tissue but marked overexpression of cyclin in glioma [23]. In our study Gaint cell, GBM had 0% expression of cyclin D1, like Homma T et al. (2006) outcome [28]. Gliosarcoma had 100% expression of cyclin D1, but the mean expression percentage is 10%. This outcome is consistent with the study done by Actor B et al. (2002) [29]. Astrocytoma is histologically divided into four grades- GradeI (pilocytic astrocytoma), Grade II (low-grade astrocytoma), Grade III (anaplastic astrocytoma), and Grade IV (Glioblastoma). The prognosis worsens with increasing grades. In our study, 72.5% of patients expired within one year of diagnosis. Work by Malmostrom A et al. showed a median survival of only three months in untreated patients [30]. As per a study done by Kleihues P et al., less than half of the patients of GBM survive for more than a year [31]. Survival analysis for subjects with different Cyclin D1 expression strata revealed that though the Median survival time was higher among those with no Cyclin D1 expression (259.00±102.21 days) as compared to those with Cyclin D1 expression (217.00±6.79 days) yet the difference between the two strata was not significant statistically (p=0.120 - Log Rank - Mantel-Cox test). But However, as compared to those who expired, the mean expression percentage was significantly higher among those who survived (p=0.035).

Similar to the study done by Tan PG et al. (2004), the cyclin D1 positive ratios were 66.67% (14/21) and 32.00% (8/25) in the dead group and survival group, respectively [14]. There was a significant difference between the two groups (P< 0.05). The higher the cyclin D1 expressed, the worse prognosis the patients had. The expression of cyclin D1 can act as a biological marker in evaluating the malignancy of gliomas and the prognosis of patients. One of the critical limitations of this study is the number of fewer cases, and an increased number of cases could have a statistical contribution. The follow-up period of all patients should be at least two years from therapy.

## Conclusions

Most patients expired during the study period, within one year of diagnosis. No significant association between Cyclin D1 expression status and different demographic, clinical, and outcome variables could be observed. Survival analysis for subjects with different Cyclin D1 expression strata revealed that the difference between the two strata was not significant. There was no association between age, gender, and diagnostic entities with cyclin D1 expression. The mean immuno-expression of Cyclin D1 was significantly higher among those who expired than those who survived. ROC curve indicated a moderate efficacy of the immuno-expression of Cyclin D1 to predict the outcome expiry. A maximum number of glioblastoma patients were aged between the fourth and fifth decades. There is male predilection. Most patients were GBM alone, and the temporal site was the most involved, followed by the parietal.
